# Assessment of drug-related problems and associated factors among patients with chronic obstructive pulmonary disease in Dailekh, Nepal: a cross-sectional study

**DOI:** 10.1038/s41598-026-51951-8

**Published:** 2026-05-12

**Authors:** Lok Raj Pant, Prakash Adhikari, Vishnu Khanal, Nim Bahadur Dangi

**Affiliations:** 1 Pharmaceutical Sciences Program, School of Health and Allied Science, Faculty of Health Sciences, Pokhara University, Pokhara-30, Kaski, Nepal; 2 Health Service Office Dailekh, Ministry of Social Development, Dailekh, Karnali Province Nepal; 3 Central Department of Public Health, Institute of Medicine, Tribhuvan University, Kathmandu, Nepal; 4 Remote Health System and Climate Change Centre, Menzies School of Health Research, Charles Darwin University, Alice Springs, Australia

**Keywords:** COPD, Drug related problems, Pulmonary disease, Nepal, Diseases, Health care, Medical research, Risk factors

## Abstract

Chronic Obstructive Pulmonary Disease (COPD) is a major health concern in low and middle-income countries. Drug-related problems (DRPs), defined as issues in drug therapy that interfere with desired outcomes, add further challenges. Evidence on the prevalence and costs burden of DRPs in COPD patients is limited. This study assessed the prevalence of DRPs, identified associated factors, and compared prescription costs between patients with and without DRPs. A cross-sectional study was conducted among 156 COPD patients attending a first referral hospital in Dailekh, Nepal, from May to September 2024. Data were collected through in-person interviews using a structured questionnaire and medical record review. DRPs were classified using the Pharmaceutical Care Network 9.1 system. Prevalence and medications costs were reported. Factors associated with DRPs were analyzed using bivariate tests and multivariate logistic regression. Medication costs were calculated using hospital pharmacy prices and mean costs with 95% confidence interval were compared across groups. The prevalence of DRPs was 71.8%, with patients experiencing an average of 1.45 DRPs. Male patients (AOR 3.0; 95% CI 1.2–7.4), those from disadvantaged ethnic group (AOR 3.3; 95% CI 1.1–9.8), and patients with comorbidity (AOR 2.7; 95% CI 1.2–5.9) were more likely to have DRPs. The mean cost per patient was NRs. 6543 ± 1423.4). DRPs are highly prevalent among COPD patients and contribute to significant financial burden. Strengthening the role of pharmacists and other health professionals in identifying and preventing DRPs is essential. Targeted interventions for high-risk groups can help reduce DRPs, improve treatment outcomes, and lessen the economic impact on patients.

## Background

Chronic Obstructive Pulmonary Disease (COPD) is a progressive lung disorder characterized by irreversible airflow obstruction caused by bronchitis and emphysema^1^. Globally, COPD affects 5–10% of individuals over 40, making it more common than asthma^2^, and it was the third leading cause of death in 2019, with 3.23 million deaths^3^. The World Health Organization reports 90% of COPD deaths occur in low-and middle-income countries^4^. Key risk factors include tobacco smoke, occupational dust, indoor and outdoor air pollution, recurrent respiratory infections, and genetic factors such as alpha-1 antitrypsin deficiency^5^. COPD is the second leading cause of death in Nepal^6^. The national prevalence is 11.7%^7^, while Karnali province, the setting of this study, shows a higher prevalence of 25.1%^8^. Management of COPD relies on maintenance pharmacotherapy to reduce symptoms, prevent exacerbations, and improve quality of life. Although COPD cannot be cured, appropriate medication helps patients manage symptoms and avoid flare-ups. However, non-adherence to medication and poor self-care remain major challenge in Nepal^9,10^. A study from Western Nepal reported that 63% of COPD patients had poor adherence to recommended medication^9^, while another study highlighted inadequate self-care^11^. Ineffective inhaler use and increasing polypharmacy further contribute to medication-related problems and hospital admissions^5^.

A drug related problem (DRP) is an event or circumstance involving drug therapy that actually or potentially interferes with desired health outcomes^12^. DRPs include unnecessary, inadequate or ineffective drug treatment, adverse drug event, inappropriate dosage, and poor adherence^12^. They impose a substantial burden on patients, communities and health systems by increasing the duration of illness, casing additional complications, raising treatment costs, and reducing productivity^13^. DRP also increases morbidity, mortality, and overall health expenditure^14^. Although DRPs are common, many are preventable. A meta-analysis by Beijer et al. found that up to 88% of adverse drug reactions in older adults, and 24% in younger individuals, were preventable^15^.

Globally, medication errors cost an estimated at US$ 42 billion annually, nearly 1% of total health spending^16^. DRPs increases hospital admission^17^, length of hospital stay, loss of productivity^18^ and psychological distress^19^. While DRPs occur in both primary and tertiary care setting^20^, estimating their burden remains challenging. Only a few studies from Asia have assessed DRPs among inpatients with COPD. A study from China reported a prevalence of 56.7%^21^. In Nepal, DRPs have been studied only in general patient population and among individuals with diabetes, with prevalence rates of 77.4%^22^ and 53.3%^23^. Awareness of DRPs remains limited, reporting is not prioritized, and the health system lacks mechanisms for routine monitoring^24^. Previous Nepalese studies have examined DRPs^22,23^, in non-communicable diseases, and they have not focused on COPD. Although COPD management guidelines are included in the PEN-plus clinical protocol of the epidemiology and disease control division, the protocol does not address how to identify or manage DRPs or guide pharmacists in this process^25^. The economic burden of DRPs is also a concern, yet no studies in Nepal have evaluated their associated costs. To address these gaps, this study studied the prevalence of DRPs, associated factors, and medication costs related to DRPs among COPD patients in a primary hospital in Karnali Province.

## Method

### Study design, population and setting

A cross-sectional study was conducted among COPD patients at Dailekh District Hospital, Karnali Province, Nepal, from May to September 2024. The hospital is located in the mid-hill region of remote Nepal and serves as the referral center for 11 municipalities with a total population of 252,313^26^. The district has seen a steady rise in COPD cases in recent years. In 2023/24, 2,812 COPD cases were reported in outpatient departments across Dailekh, with 41.3% (*n* = 1162) presenting at Dailekh District Hospital, an increase from 23.9% in 2022/23^27^. Communities in the study area experience poor housing conditions (91.8% mud-bonded walls), high indoor air pollution, and widespread use of polluting fuels (89.6% firewood), all of which contribute to elevated COPD risk^26^.

### Selection of participants

A purposive sampling method was used due to the low volume of COPD admissions in rural hospitals and to ensure feasibility. Participants were patients admitted to the General In-Patient Unit who were 18 years or older, able to communicate, and had an initial diagnosis of acute exacerbation of COPD while receiving COPD medications. Patients unable to respond due to severe illness, those unwilling to provide informed consent, or those with severe cognitive or psychiatric conditions were excluded. Pregnant women were also excluded because their treatment regimen differs from standard COPD management. A total of 156 participants were enrolled. The sample size was determined based on an estimated COPD prevalence of 11.5%^28^ and a maximum allowable error of 5%. Each participant was interviewed using a structured questionnaire, and medication profiles were reviewed. Drug-related problems (DRPs) were identified using the Pharmaceutical Care Network Europe (PCNE) classification version 9.1.

### Variables

#### Outcome variables

Two key outcome variables were: (i) drug related problems and (ii) medication related costs. Drug related problems were described as an event or circumstance involving drug therapy that actually or potentially interferes with desired health outcomes. It was measured as defined by PCNE v9.1^11^. A pharmacist conducted daily reviews to identify DRPs from the information of each participant’s drug therapy; collaborating with physicians and nurses from admission to discharge. When causes were identified, related potential problems were also recorded, including both actual and potential issues per the PCNE standards^11^.

Medication related cost was measured by calculating the cost of medications prescribed by healthcare practitioners. Medication related cost was determined using the maximum retail price of drugs by the hospital. The cost data were obtained from the hospital pharmacy’s official purchase list for fiscal year 2023/24. All estimates were made in Nepalese Rupees (NRs; 1USD = 140.2 NRs). Our estimate does not include the health system and personnel cost.

#### Independent variables

Key independent variables included Sociodemographic, health history data and community specific data. Age, gender (categorized as male, female and others) which was later categorized only as male and female due to absence of others group. Ethnicity was classified according to the health management information system classification of Nepal (Dalit, Janajati, Madhesi, Muslim, Brahmin/Chhetri and Others). Ethnicity was further categorized into Dalit/Janajati and Brahmin/Others to facilitate the analysis. Lifestyle-related factors such as habit of drinking alcohol and smoking were also included, with each categorized as never used, current user and previous user. For further analysis they were categorized as never used and ever used (current and previous smokers). Place of residence were bifurcated into urban and rural categories (metropolitan, sub-metropolitan, and urban municipalities constituted the urban group, whereas rural municipalities represented the rural cohort). Educational level was categorized into no formal education and formal education. Additionally, the presence or absence of parental history of COPD was recorded as present (if answered yes), absent (if answered no) and unknown (if not the respondents did not know).

Clinical characteristics were measured through patient medical history review; the comorbidity status was categorized as present and absent. The duration of COPD diagnosis was measured in years. The severity of the COPD was recorded as mild, moderate and severe based on the severity classification. However, mild had zero observations therefore, only moderate and severe were included as these patients were admitted to hospital. Drug interaction was recorded as present and absent using Medscape database, Micromedex mobile Version 3.0.6(3063) and UpToDate website was used. The length of hospital stay was measured in days of admission to discharge. Drugs prescribed were measured in terms of their count.

### Tool and technique of data collection

A structured questionnaire and standard tools were used for data collection. Pretesting was conducted among 16 patients. Face-to-face interviews were conducted with all eligible participants. The DRP assessment was performed independently and in a blinded manner without involvement of the treating physicians. DRPs were identified using the PCNE 9.1. The PCNE V9.1 classification system is a validated tool used to identify and document drug-related problems by categorizing them into problems and causes. The ‘problems’ domain focuses on clinical outcomes, particularly treatment effectiveness and patient safety, while the ‘causes’ domain explores the underlying reasons for these issues, including inappropriate drug selection, dosage form errors, dispensing failures, and patient non-adherence. PCNE V9.1 has also been previously used in Nepal to assess drug-related problems^12^.

Two trained pharmacy professionals independently reviewed medication profiles. Inter rater reliability was assessed during the pre-testing phase, yielding a Cohen’s kappa of 0.70, indicating substantial agreement. Discrepancies were resolved through discussion and consensus using the PCNE manual as reference. Cost data were collected using a structured form that recorded the medications purchased by each patient. Medication costs were calculated using the hospital’s maximum retail price list and patients’ medicine purchase bills. Only the direct cost of medicines and surgical items, including medical devices such as nebulizer masks, Zerostat, masks, spirometers, and other assistive devices, was considered. Other healthcare-related expenses were excluded.

### Data analysis

The prevalence of DRPs was reported using frequency distributions. Associations between DRPs and independent variables were first explored using the Chi-square test, followed by multiple logistic regression analysis. Variables with a p-value < 0.10 in the bivariate analysis were included in the multivariate model. Variables with cell counts less than 5 in the chi-square test were analyzed using Fisher’s exact test, as their confidence intervals were high and they were not included in the multivariate analysis. The cost of prescription medications for COPD was reported as mean values with 95% confidence intervals and standard deviation to provide a descriptive comparison of treatment costs between patients with and without DRPs, highlighting the potential economic burden associated with DRPs. Statistical significance for the multivariate logistic regression was set at *p* < 0.05. Data analysis was performed using SPSS version 25.

## Results

### Participant characteristics

A total of 156 participants were included in the study, with a mean age of 72.2 ± 9.3 years. More than half (55.8%) were female. Most participants (73.1%) had never consumed alcohol, and 30.8% had never smoked. Only 14.7% had received formal education. About 23.1% reported a parental history of COPD. Two-thirds of the patients (67.3%) had at least one comorbidity. The average duration since COPD diagnosis was 9.0 ± 5.1 years. Severe COPD was identified in 7.1% of the patients. Nearly half (44.9%) experienced drug-drug interactions. The mean length of hospital stay was 3.4 ± 0.7 days, and an average of 9.5 ± 2.2 medications was prescribed per patient (Table 1).

**Table 1:** Characteristics of the participants.

Characteristics	*n* (%)
Age (years)	Mean: 72.2 ± 9.3
Less than 65	29 (18.6%)
Younger old (65–74)	59 (37.8%)
Middle old (75–84)	55 (34.4%)
Older old (> 85)	13 (8.2%)
Gender	
Male	69 (44.2%)
Female	87 (55.8%)
Ethnicity	
Dalit/Janajati	42 (24.9%)
Brahmin/Chhetri/Others	114 (75.1%)
Alcohol consumption	
Never used	114 (73.1%)
Ever used	42 (26.9%)
Smoking	
Never used	48 (30.8%)
Ever used	108 (69.2%)
Residence	
Urban	87 (55.8%)
Rural	69 (44.2%)
Educational Level	
Illiterate/informal education	133 (85.3%)
Literate/formal education	23 (14.7%)
Parental history of COPD	
Absent	17 (10.9%)
Present	37 (23.1%)
Unknown	102 (66.0%)
Comorbidity	
Present	105 (67.3%)
Absent	51 (32.7%)
Duration of COPD diagnosis (years)	9.0 ± 5.1
< 5	26 (16.7%)
5–9	56 (35.9%)
10–14	48 (30.7%)
≥ 15	26 (16.7%)
Severity of COPD	
Moderate	145 (92.9%)
Severe	11 (7.1%)
Drug interaction	
No interaction	86 (55.1%)
Interaction	70 (44.9%)
Length of hospital stay (in days)	Mean: 3.4 ± 0.7
Number of drugs prescribed	Mean: 9.5 ± 2.2

### Drug related problems identified

A total of 226 identified DRPs were grouped into three main domains. Problems related to Treatment Effectiveness (P1) made up the largest share at 59.3%, followed by Treatment Safety (P2) at 28.3%, and other problems at 12.4% (Table 2). About one-quarter of patients had one DRP (26.1%) and a similar proportion had two DRPs (26.9%). Only a small group (5.13%) reported three or more DRPs.

**Table 2:** Drug related problems identified.

Primary domain (code)	Sub domain (problems with code)	*n* (%)
Treatment effectiveness (P1)	No effect of drug treatment despite correct use (P1.1)	28 (12.4)
	Effect of drug treatment not optimal (P1.2)	78 (34.5)
	Untreated symptoms or indication (P1.3)	28 (12.4)
Treatment safety (P2)	Adverse drug event (possibly) occurring (P2.1)	64 (28.3)
Other problems (P3)	Unnecessary drug treatment (P3.1)	14 (6.2)
	Unclear problem/complaint (P3.2)	14 (6.2)
Total problems		226 (100)

### Causes of DRPs

A total of 344 causes of drug-related problems were identified, averaging 2.2 causes per patient. Patient-related factors were the most frequent, accounting for 40.7% of all causes. Issues related to drug selection made up 16.9%, while dispensing problems accounted for 27.9%. Dose-selection issues contributed 5.5% of the causes (Table 3).

**Table 3:** Associated factors of DRPs.

Primary domain (code)	Sub domain (causes with code)	*n* (%)
Drug selection (C1)	Inappropriate drug according to guidelines/formulary (C1.1)	11 (3.2)
	No indication of drug (C1.2)	1 (0.3)
	Inappropriate combination of drugs, drugs and herbal medications, or drugs and dietary supplements (C1.3)	24 (7.0)
	Inappropriate duplication of the therapeutic group or active ingredient (C1.4)	4 (1.2)
	No or incomplete drug treatment despite existing indication (C1.5)	7 (2.0)
	Too many different drugs/active ingredients prescribed for indication (C1.6)	11 (3.2)
Dosage form (C2)	Inappropriate drug form/formulation (for this patient) (C2.1)	4 (1.2)
Dose selection (C3)	Drug dose too low instructions wrong, unclear or missing (C3.1)	9 (2.6)
	Drug dose of a single active ingredient too high (C3.2)	4 (1.2)
	The dosage regimen is not frequent enough (C3.3)	1 (0.3)
	Dose timing (C3.5)	5 (1.5)
Treatment duration (C4)	Duration of treatment too short (C4.1)	2 (0.6)
	Duration of treatment too long (C4.1)	3 (0.9)
Dispensing (C5)	Prescribed drugs not available (C5.1)	57 (16.6)
	Necessary information not provided or incorrect advice provided (C5.2)	37 (10.8)
	Wrong drug or strength dispensed (C5.4)	2 (0.6)
Drug use process (C6)	Inappropriate timing of administration or dosing intervals by a health professional (C6.1)	8 (2.3)
	Drug under-administered by a health professional (C6.2)	2 (0.6)
	Drug over-administered by a health professional (C6.3)	3 (0.9)
	Drugs are not administered at all by a health professional (C6.4)	2 (0.6)
	Drug administered via the wrong route by a health professional (C6.6)	2 (0.6)
Patient related (C7)	The patient intentionally uses/takes less drug than prescribed or does not take the drug at all for whatever reason (C7.1)	26 (7.6)
	The patient uses/takes more drugs than prescribed (C7.2)	11 (3.2)
	Inappropriate timing or dosing intervals (C7.7)	17 (4.9)
	The patient unintentionally administers/uses the drug in the wrong way (C7.8)	53 (15.4)
	The patient is physically unable to use the drug/form as directed (C7.9)	10 (2.9)
	The patient is unable to understand instructions properly (C7.10)	23 (6.7)
Others (C9)	No or inappropriate outcome monitoring (incl. TDM) (C9.1)	2 (0.6)
	Other causes; specify (C9.2)	1 (0.3)
	No obvious cause (C9.3)	2 (0.6)
Total cause		344 (100)

We have only included and quantified the identified causes.

### Factors associated with the presence of DRPs

Drug-related problems were more common among older patients, males, and those belonging to Dalit or Janajati groups (Table 4). Individuals without formal education and those with comorbidities also showed a higher presence of DRPs. All patients with severe COPD had DRPs, and almost every patient with documented drug interactions experienced at least one DRP. Other factors, including alcohol use, smoking status, residence, and family history of COPD, showed no meaningful differences. Table 5 shows the results of the multiple logistic regression analysis. Patients who were male (AOR 3.0; 95% CI 1.2–7.4), from Dalit or Janajati groups (AOR 3.3; 95% CI 1.1–9.8), and those with comorbidities (AOR 2.7; 95% CI 1.2–5.9) had a significantly higher likelihood of experiencing DRPs compared to their counterparts.

**Table 4:** Distribution of DRPs by patient characteristics.

Characteristics	DRP present	DRP absent	*p*-value
Age			
Less than 65 years	17 (58.6)	12 (41.4)	0.08
65 and above	95 (74.8)	32 (25.2)	
Gender			
Male	55 (79.7)	14 (20.3)	0.05
Female	57 (65.5)	30 (34.5)	
Ethnicity			
Dalit/Janajati	37 (88.1)	5 (11.9)	0.01
Brahmin/others	75 (65.8)	39 (34.2)	
Alcohol drinking			
Never used	80 (70.2)	34 (29.8)	0.46
Ever used	32 (76.2)	10 (23.8)	
Smoking			
Never used	32 (66.7)	16 (33.3)	0.34
Ever used	80 (74.1)	28 (25.9)	
Residence			
Urban	63 (72.4)	24 (27.6)	0.84
Rural	49 (71.0)	20 (29.0)	
Educational level			
No formal education	100 (75.2)	33 (24.8)	0.02
Formal education	12 (52.2)	11 (47.8)	
Family history of COPD			
Known history	38 (70.4)	16 (29.6)	0.77
Unknown history	74 (72.5)	28 (27.5)	
Comorbidity			
Present	28 (54.9)	23 (45.1)	0.01
Absent	84 (80)	21 (20)	
Severity of disease*			
Moderate	101 (69.7)	44 (30.3)	0.03
Severe	11 (100.0)	0 (0.0)	
Drug interaction*			
No interaction	43 (50)	43 (50)	0.01
Interaction	69 (98.6)	1 (1.4)	
Total cost of prescribed drug (NRs)^#^			
≤ median	55 (66.3)	28 (33.7)	0.10
> median	57 (78.1)	16 (21.9)	

*Cell value < 5 and were excluded from regression analysis; ^#^median cost: NRs 6790.

**Table 5:** Factors associated with the presence of DRPs.

Characteristics	Adjusted OR (95% CI)	*p*-value
Gender		
Female	1.0	0.02*
Male	3.0 (1.2–7.4)	
Ethnicity		
Brahmin/others	1.0	0.03*
Dalit/Janajati	3.3 (1.1–9.8)	
Comorbidity		
Absent	1.0	0.01*
Present	2.7 (1.2–5.9)	
Age		
65 and above	1.0	0.26
Less than 65 years	0.59 (0.23–1.49)	
Education		
Formal education	1.0	0.07
No formal education	2.83 (0.92–8.73)	

### The cost of prescribed drugs

The average cost of prescribed drugs per patient was NRs 6543.0 (95% CI 6317.8–6768.2; SD 1423.9). Patients with at least one DRP incurred a slightly higher mean cost (NRs 6678.8; 95% CI 6413.9–6943.8) compared to those without DRPs (NRs 6197.3; 95% CI 5770.7–6623.9). Among DRP causes, the highest mean cost was observed in patients with issues related to the drug use process (C6: NRs 7032.9; 95% CI 6368.0–7697.9), while the lowest was seen in patients with dosage form issues (C2: NRs 6096.7; 95% CI 4565.3–7628.1). No statistically significant differences were observed in the mean costs between patients with and without DRPs across the various DRP classifications (Table 6).

**Table 6:** Mean cost incurred among the patients with and without DRPs.

Description	Mean cost (95% CI) in NRs	
	DRP absent	DRP present
Average cost of a patient: NRs 6543.0: (95% CI NRs 6317.8, 6768.2)		
Cost estimate by number of DRPs		
At least one problem/cause	6197.3 (5770.7, 6623.9)	6678.8 (6413.9, 6943.8)
Problem 1 (treatment effectiveness)	6396.0 (6014.0, 6777.4)	6618.6 (6336.2, 6901.1)
Problem 2 (treatment safety)	6431.0 (6140.0, 6721.9)	6704.1 (6342.7, 7065.4)
Problem 3 (others)	6578.1 (6338.1, 6818.1)	6350.0 (5675.7, 7024.3)
Cost estimate by DRP classification		
C1 Drug selection	6437.7 (6175.1, 6700.2)	6811.1 (6367.3, 7254.9)
C2 Dosage form	6551.7 (6322.6, 6780.9)	6096.7 (4565.3, 7628.1)
C3 Dose selection	6253.6 (6279.6, 6767.6)	6691.7 (6076.7, 7306.7)
C4 Treatment duration	6546.5 (6317.1, 6775.7)	6458.3 (4746.0, 8170.7)
C5 Dispensing	6287.1 (5963.6, 6610.6)	6812.4 (6504.0, 7120.7)
C6 Drug use process	6483.1 (6242.3, 6723.2)	7032.9 (6368.0, 7697.9)
C7 Patient related	6374.9 (6064.4, 6685.3)	6687.1 (6361.8, 7012.5)
C9 Others	6558.0 (6327.4, 6788.5)	6092.0 (4706.8, 7477.2)

C8: Patient transfer related cause was not measured as it was based on signal setting. DRP, Drug related problem, NRs, Nepali rupee, CI, Confidence interval.

## Discussion

This study found the prevalence of DRPs, their associated factors, and the medication costs associated to DRPs among COPD patients in a district hospital of Karnali Province, Nepal. The findings show a high burden of DRPs, with 71.8% of patients experiencing at least one DRP, an average of 1.5 DRPs per patient, and 2.2 causes per patient.

The prevalence observed in this study is comparable to previous studies from Nepal, which reported DRP prevalence of 77.4% in general wards and 74.2% in ICU/HCU settings^22,29^. The high prevalence may reflect gaps in documentation, monitoring, and the limited availability of pharmacy personnel. Although the Hospital Pharmacy Guideline 2015 of Nepal recommends one pharmacist and three assistant pharmacists for hospitals with 26–50 beds^30^, the study hospital had only one assistant pharmacist. Evidence consistently shows that active pharmacist involvement improves treatment outcomes and overall disease management^39^. Treatment effectiveness issues were the most common DRP (59.3%), consistent with findings from Nepal^22^ and Turkey^31^. In contrast, studies from China identified treatment safety as the predominant issue^21^. These differences may relate to variations in healthcare systems, monitoring practices, and the availability of trained clinical pharmacists, who play a key role in DRP identification and medication reconciliation^32^.

Patient related causes accounted for the highest proportion of DRP causes (40.7%), followed by dispensing issues (27.9%) and drug selection problems (16.9%). In Nepal, patient related factors such as financial constraints, reliance on alternative therapies, and inadequate adherence remain common^9^. This aligns with findings from Ethiopia, where non-adherence was also frequently reported^33^. In contrast, drug selection issues were more prominent in studies from China and Eastern Nepal^22^, likely reflecting the complexity of prescribing in tertiary hospitals. The absence of structured counseling services, lack of dedicated spaces, limited availability of qualified pharmacists, and the absence of clinical pharmacy services likely contribute to these issues. Dispensing problems were often linked to stockouts caused by procurement inefficiencies, affecting the availability of medications such as Digoxin, Amiodarone, and various inhalation capsules^34^. These findings highlight the need for evidence-based prescribing, guideline adherence^35^, and tools such as the Beers criteria to minimize medication-related risks^36^. Three factors gender, ethnicity, and comorbidities were significantly associated with DRPs. Male patients were more likely to experience DRPs, consistent with findings from ICU/HDU settings in central Nepal^29^. Differences in health-seeking behavior, adherence patterns, and pharmacokinetic variations across genders may partly explain this trend. Higher alcohol use among men may also contribute through alcohol–drug interactions^37^. These observations underscore the need for gender-sensitive patient education and monitoring.

Dalit and Janajati patients were more likely to experience DRPs, a pattern supported by a systematic review by Baehr et al.^38^. Financial barriers to care^39^, low health literacy^40^, and poorer adherence^41^ likely contribute to these disparities. Culturally tailored interventions and further research across diverse socio-economic groups are needed to better understand and address these gaps. Comorbidities were also strongly associated with DRPs, consistent with previous evidence from Nepal^22^. Managing multiple chronic conditions increases the likelihood of polypharmacy^42^, drug interactions^43^, and regimen complexity. With the growing burden of chronic diseases in the elderly^44^, polypharmacy has significant impacts on health outcomes^45^. Simplifying medication regimens and strengthening clinical monitoring for patients with multi morbidity are essential steps. The mean hospital pharmacy cost per inpatient (NRs. 6543) was higher than the annual health insurance premium for a family of five (NRs. 3500)^46^, indicating that health insurance provides meaningful financial protection, even in primary care settings. No significant difference in pharmacy costs was observed between patients with and without DRPs. Because this study was conducted in a remote primary hospital where high-cost interventions are rare, cost patterns may differ from urban or private-sector hospitals, and this should be considered when comparing results.

To our knowledge, there are very few studies in South Asia to assess DRPs among COPD patients in a primary care setting using the PCNE v9.1 classification. The study has some limitations, including a modest sample size, single-site design, exclusion of intervention-related variables, and reliance on hospital pharmacy cost data, which may underestimate total expenditures. Seasonal variations were also not captured. Despite these limitations, the findings offer important insights for future research and for designing interventions to reduce DRPs. Overall; the study underscores the need for a multidisciplinary approach to improve medication safety. Gender-sensitive and culturally appropriate strategies, along with strengthened pharmacist involvement, are essential to reducing DRPs and improving treatment outcomes in Nepal.

## Conclusion

This study demonstrates a high burden of DRPs among COPD patients, with nearly three-quarters experiencing at least one DRP. Gender, ethnicity, and comorbidities were significantly associated with the presence of DRPs. No meaningful difference in medication cost was found between patients with and without DRPs. Interventions that address gender-related needs, cultural differences, and the challenges of managing comorbid conditions are essential. Strengthening the role of pharmacists in monitoring prescriptions, supporting medication use, and improving health literacy on DRPs is equally important. Further action-oriented research is needed to identify practical strategies for reducing DRPs within low-resource healthcare settings.

## Data Availability

All data are fully available without restriction.
